# Peroral Endoscopic Myotomy Can Improve Esophageal Motility in Patients with Achalasia from a Large Sample Self-Control Research (66 Patients)

**DOI:** 10.1371/journal.pone.0125942

**Published:** 2015-05-18

**Authors:** Shuangzhe Yao, Enqiang Linghu

**Affiliations:** Department of Gastroenterology, Chinese People's Liberation Army General Hospital, No. 28, Fuxing Road, Haidian District, Beijing 100853, China; University Hospital Llandough, UNITED KINGDOM

## Abstract

**Background:**

Peroral endoscopic myotomy (POEM) as a new approach to achalasia attracts broad attention. The primary objective of this study was to evaluate the results with esophageal motility after POEM through the first large sample clinical research.

**Patients and Methods:**

We have a self-control research with all patients (205 in total) who underwent POEM from 2010 to 2014 at our Digestive Endoscopic Center, 66 patients of which underwent high resolution manometry (HRM) before and after POEM in our motility laboratory. Follow-ups last for 5.6 months on average. Outcome variables analyzed included upper esophageal sphincter pressure (UESP), upper esophageal sphincter residual pressure (UESRP), lower esophageal sphincter pressure (LESP), lower esophageal sphincter residual pressure (LESRP) and esophageal body peristalsis. We have a statistical analysis to illustrate how POEM impacts on the change of esophageal motility.

**Results:**

The symptoms related to dysphagia were relieved in 95% of patients in recent term after POEM. While HRM showed a statistically significant reduction of URSRP, LESP and LESRP (P<0.01), however, peristalsis was not consistently affected. There were 11 patients who had undergone other prior endoscopic treatment (endoscopic dilation or botulinum toxin injection) and 55 patients had not. The statistical difference (P>0.05) did not occur for these two groups on LESP and LESRP reduction.

**Conclusions:**

POEM clearly relieved the symptoms related to dysphagia by lowering the pressure of upper esophageal sphincter (UES) and lower esophageal sphincter (LES),and other endoscopic treatment before POEM did not affect the improvement of LES pressure. These results are concluded from our short-term follow-up study, while the long-term efficacy remains to be further illustrated.

**Trial Registration:**

Chinese Clinical Trial Register ChiCTR-TRC-12002204)

## Introduction

Achalasia is a neurodegenerative motility disorder of the esophagus resulting in absence of peristalsis on esophageal body and impaired relaxation of lower esophageal sphincter(LES) in response to swallowing. At present,all therapis can not treat the underlying problem because the etiology of achalasia is not yet clear.The method of incision LES whether endoscopic or surgical interventions can reduce the pressure of LES in aid of esophageal emptying just by damaging the integrality of anatomy. In 2010, peroral endoscopic myotomy (POEM) was reported clinically by Inoue and colleagues for the first time[1],the symptom remission rate of 93–100%[2–6] made the procedure an attractive primary therapeutic option for the patients with achalasia.

Several studies have reported that POEM was safe and effective in treating achalasia, however, the complications of POEM, both short term and long term, was reported in range from 11.8% to 100%[1–6]. So far, we have no clinical research on large sample that evaluate the results after POEM by esophageal motility.

In the current study, we arrange our experience with POEM for the treatment of achalasia and the effect on esophageal motility.

## Patients and Methods

### Patients

As a self-control study, all patients signed the consent form.

**Fig 1:**
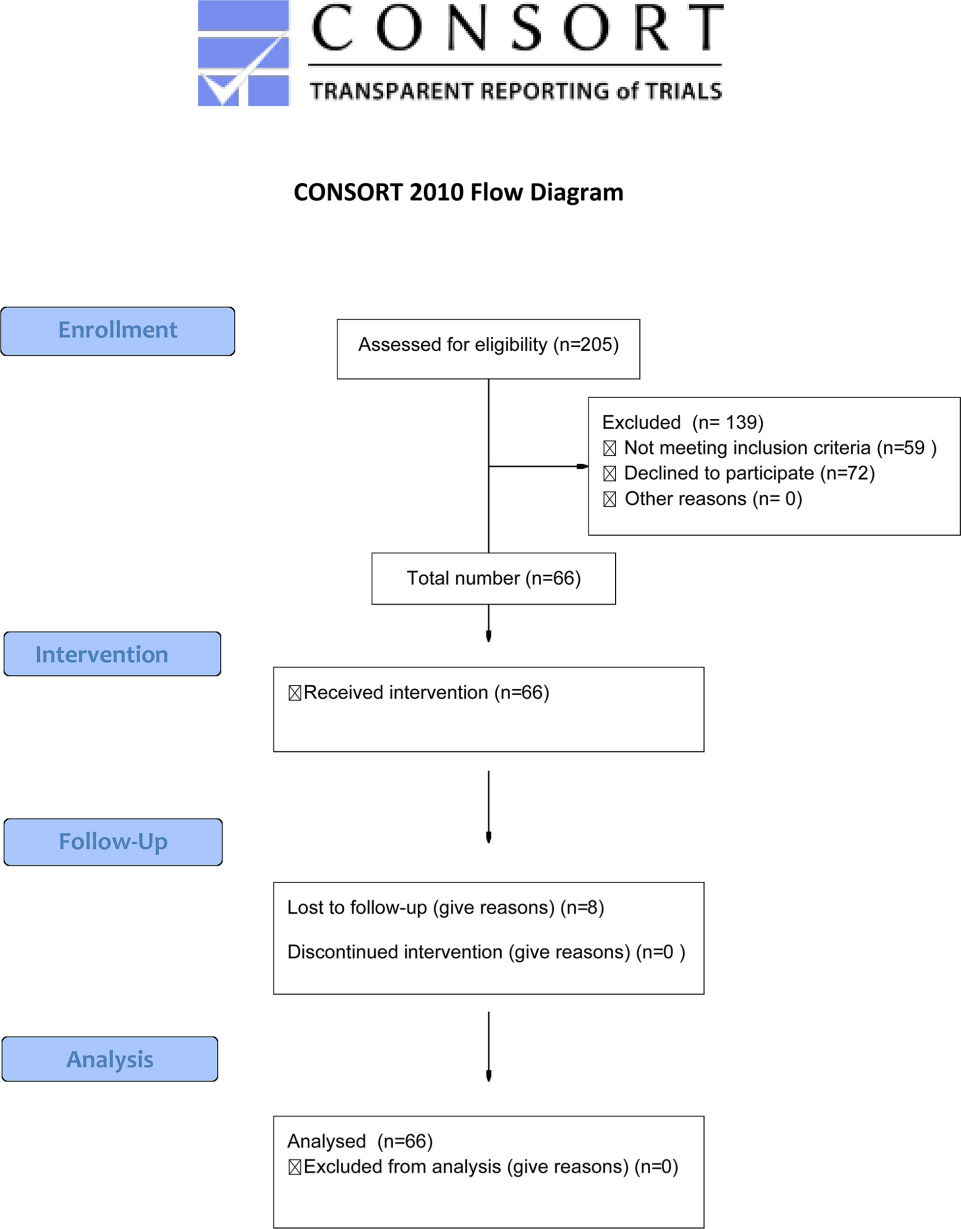
Research Flow Diagram.

We screened out 205 patients with achalasia diagnosed definitely and then underwent POEM from December 2012 to April 2014 at our Digestive Endoscopic Center by Linghu EQ. Exclusion criteria included previous Heller myotomy in surgery, malignant or pre-malignant esophageal lesions (Fig 1). The protocol for this trial and supporting TREND checklist are available as supporting information; see S1 Checklist and S1 Protocol.

Among them, 59 patients had high resolution manometry(HRM) in other Digestive Endoscopic Center, while 72 patients did not give a permission to perform HRM before POEM because of price and necessity. As a result, 74 patients have integrated HRM before and 66 patients have also after POEM. There were 34 male and 32 female patients with a mean age of 44.6 years (range 14 to 76). Mean follow-up period was 5.6 months (Table 1).

**Table 1:** Patient Characteristics.

Characteristic	Data
Age,mean(range),y	44.6(14–76)
Sex,No.male/female	34/32
Duration of symptoms,mean(range),y	5(0.3–30)
Duration of follow-up,mean(range),m	5.6(1–12)
Achalasia Ling Subtypes,No	
Type I	27
Type II	35
Type III	4
Prior endoscopic treatment,No.yes/no	11/55

Chinese Clinical Trail Registry and Committee of medical ethics of the Chinese PLA General Hospital approved our research and consent procedure. All participants provide their written informed consent to participate in this study.

### Outcome measurements

Upper esophageal sphincter pressure (UESP) and lower esophageal sphincter pressure (LESP) were calculated as the mid-expiratory pressure at the respiratory inversion point. Upper esophageal sphincter residual pressure (UESRP) and lower esophageal sphincter residual pressure (LESRP) were defined as the minimum pressure recorded in the LES during swallowing.

### POEM procedure

Based on their endoscopic characterizations, 27 patients had Ling I achalasia, 35 patients had Ling II achalasia and 4 patients had Ling III achalasia[7]. As we know, the procedure of POEM can be generally divided into four steps, including entry incision on mucosa, establishment of the tunnel on submucosa, myotomy and sealing of the entry incision.

### Esophageal manometry

HRM was performed by using the following protocol: a 36-channel, solid-state catheter system with high-fidelity circumferential sensors at 1-cm intervals was advanced through the nasal canal (Manosacn; Sierra Scientific Instruments Inc, Los Angeles, CA). Studies were performed in a supine position after at least a 6-hour fast. Pressure data of 10 wet swallows was recorded and analyzed by using a dedicated computerized analysis system. All relevant parameters were calculated according to the Chicago classification.

### Data analysis and statistical analysis

Data were compared before and within 1 year after POEM. Statistical analysis was performed by using SPSS 16.0. Data are presented as mean ± standard error of the mean when parametric. Statistical analysis included paired t-test, analysis of variance, and univariate regression analysis (Pearson). All reported *P* values are 2 tailed. A probability of less than 5% was assumed to be statistically significant (*P* < 0.05).

(I affirm that all authors had access to the study data and reviewed and approved the final manuscript.)

## Results

### Patients

Mean duration of symptoms for the 66 patients with achalasia diagnosed definitely was 5 years. 11 patients (16.7%) had received endoscopic treatment before. 7 patients underwent up to balloon dilation and 4 patients was treated with botulinum toxin injection. 32 patients (48.5%) had the myotomy for circular muscles individually and 34 patients (51.5%) had the myotomy of circular muscles and longitudinal muscles.

### POEM procedure

The POEM procedure was safely performed in all patients. No technical difficulties occurred perioperatively. All patients were discharged after 7 days of hospitalization.

### Remission of symptoms

The symptoms related to dysphagia were relieved in 63 patients (95%) in recent term after POEM. Most patients just can take liquid diet pre-POEM, and then can take full diet pro-POEM.

### Upper esophageal sphincter (UES) pressure based on HRM

HRM was performed in 66 patients pre and post POEM in our motility laboratory. Mean pre-POEM UESP was 61.618 mmHg, with a standard deviation (SD) of 23.628, while mean post-POEM UESP was 57.403 mmHg with an SD of 22.911 (*P* > 0.05). In addition, mean pre-POEM UESRP was 16.468 mmHg, with a SD of 21.672, while mean post-POEM UESRP was 8.982 mmHg with an SD of 7.601 (*P* < 0.001) (95% confidence interval of the mean difference, CI:-2.201–10.631).

### LES pressure based on HRM

Mean pre-POEM LESP was 34.678 mmHg, with a SD of 14.908, while mean post-POEM LESP was 16.612 mmHg with an SD of 8.671 (*P* < 0.001) (95% confidence interval of the mean difference, CI:13.324–18.967). In addition, mean pre-POEM LESRP was 27.459 mmHg, with a SD of 10.719, while mean post-POEM LESRP was 11.313 mmHg with an SD of 6.792 (*P* < 0.001) (95% confidence interval of the mean difference, CI:14.284–21.850) (Table 2).

**Table 2:** Variables on pressure of esophagus.

Pre-POEM,mmHg		Post-POEM,mmHg	Decrease,%	*P* Value
UESP	61.618	57.403	6.84	0.194
UESRP	16.468	8.982	45.46	0.008
LESP	34.678	16.612	52.10	<0.001
LESRP	27.459	11.313	58.80	<0.001

### Esophageal body peristalsis on HRM

Antegrade peristalsis was not noted in all 66 patients pre-POEM and some degree antegrade peristalsis post-POEM in only 2 patients.

### Influence on the results of POEM by other prior endoscopic treatment

11 of 66 patients **(**16.7%) had undergone other prior endoscopic treatment (endoscopic dilation or botulinum toxin injection), while 55 of 66 patients (83.3%) undergone POEM as primary treatment. There are no significant differences (*P* > 0.05) on the reduction of LESP and LESRP respectively between these two groups.

### Influence on the results of POEM by muscular layer of myotomy

32 of 66 patients (48.5%) had the myotomy for circular muscles only and 34 patients (51.5%) had the myotomy of circular muscles and longitudinal muscles. Statistically, the differences between the two groups are not significant (P > 0.05) on the reduction of LESP and LESRP respectively.

### Influence factor on LESRP reduction percentage

There is no linear correlation emerged between age and LESP reduction percentage (r = -0.069, *p* > 0.05), and, between age and LESRP reduction percentage (r = -0.04, *p* > 0.05) with POEM. On the other hand, duration of symptoms and LESP reduction percentage (r = -0.182, *p* > 0.05), and, duration of symptoms and LESRP reduction percentage (r = -0.04, *p* > 0.05) has no linear correlation with POEM.

## Discussion

Achalasia is rare motor disorder of the esophagus, the etiology is likely to be affected by various factors, immune-mediated ganglionitis which results in degeneration of the myenteric nerve plexus of the esophageal wall. Although achalasia is a relatively rare condition, it carries a risk of complications, including aspiration pneumonia and esophageal cancer. Therefore, active and proper treatment is needed for the patients with achalasia. Present approaches used to treat achalasia destroy the LES rather than try to correct the underlying abnormality and to restore functionality. POEM as an attractive option is promoted in clinical cases.

In this study, we evaluated the effect of POEM on esophageal function parameters in patients with achalasia. This is the first large sample study that shows POEM can significantly improve esophageal motility by decreased UES and LES pressure[8]. It is worth mentioning that POEM has no effect on esophageal body peristalsis.

It can be found that POEM significantly reduced LESP (*p* < 0.001) and LESRP (*p* < 0.001) in short term. The inviolable anatomic integrity on LES was destroyed through POEM, so the incomplete relaxation of the LES was improved when swallowing after POEM. We showed that 95% of patients were successfully treated with regard to symptoms, in line with other reports (93–100%)[2–6]. Since the impaired inviolable anatomic integrity on LES, however, post-POEM gastroesophageal reflux remains a major problem (up to 46% in one study[9]), the glasses style anti-reflux myotomy, which retains about 1 cm of longitudinal muscle, is expected to achieve a best result to prevent the reflux after POEM[10].

Evaluation of esophageal function parameters is of great value because prior studies shows that it is a useful technique not only in diagnosing achalasia but also in providing valuable information on long term clinical response after treatment[11–13]. Eckardt and colleagues[14] noted that all patients with a post-procedure LESP less than 10 mmHg were in remission after 2 years, 71% were in remission for pressures between 10 and 20 mmHg, and 23% for pressures over 20mmHg. More recently, Hulselmans and colleagues[13] reported that 66% of patients with postprocedure LESP less than 15mmHg were in symptomatic remission after 6 years. In the current study, we found that 13 of our patients (19.7%) had a post-POEM LES pressure of less than 10mmHg, and 30 of our patients (45.5%) less than 15mmHg and 43 of our patients (65.2%) less than 20mmHg. From this, we can give a prediction that the symptom alleviation rate in short and medium term is excellent. However, with the formation of granulation in LES incision and the persistence of original neurogenic etiology, the recurrence rate in long term needs more follow-up studies.

We found that there is a significant reduction on UESRP (*P* < 0.01) after POEM. When food was accumulated in esophagus of patients with achalasia, as a result, the UES was contracted compensatorily in order to avoiding the food reflux while swallowing. After POEM, with the relaxation of LES, the compensatory contraction was released. Consequently, the UESRP was reduced significantly. As for the UESP, we believe the reason for no decreasing obviously (*P* > 0.05) was short duration of follow-up post-POEM, the functional recovery of UES has not yet achieved completely.

One study[15] shows that the dysfunction of the esophageal body may occur secondary to the high pressure of LES in achalasia, because the researchers found that some patients really can recover peristalsis partially after POEM. However, in our study, the impairment of peristalsis was still persisted after POEM within one year. Maybe the improvement on peristalsis is difficult to achieve by the therapies acting on LES. On the other hand, we consider that the sensitivity of visceral perception could be decreased with LES pressure relaxed, even if the impairment of peristalsis was still persisted, the dysphagia was improved after POEM. Larger studies with long-term follow-up are needed to explain these findings.

The minimally invasive treatments including endoscopic dilation and botulinum toxin injection have resulted in shorter patient hospitalization, reduced morbidity, and a quicker return to daily activities, so many centers make them preferred treatment for achalasia. However, these endoscopic treatment before laparoscopic Heller myotomy (LHM) will increase the possibility of failure if the patients underwent endoscopic treatment go for surgery later[16–18]. The current research shows that endoscopic treatment before POEM did not affect the improvement of LESP and LESRP (p > 0.05). Consequently, facing to a recurrent patient with endoscopic treatment before, if you concerned the curative effect of LHM, POEM is a better choice.

We all know that, HRM in the current format can reflect the pressure of circular muscles besides longitudinal muscles because axial contraction of longitudinal muscles does not change the pressure in esophagus. In our study, we found that there is no significant difference (*P* > 0.05) on LESP and LESRP reduction in the two group patients which had the myotomy of circular and longitudinal muscles or circular muscles only. It follows that the myotomy of longitudinal muscles did not ascend LES relaxation. What’s more, longitudinal muscles of the esophagus play an important role in the physiology of motility disorders, and recent studies suggest that longitudinal muscle contraction of the esophagus induces LES relaxation and even possibly improves peristalsis of the esophagus[19]. We regret that, the data which reflect esophageal body peristalsis exist much null value owing to large width on esophageal body, so there is no statistical analysis on peristalsis between the myotomy of circular and the myotomy of circular and longitudinal muscles. Regarding the role of longitudinal muscles and circular muscles for achalasia, more studies are required.

In addition, according to our study, age and duration of symptoms on patients with achalasia has no linear correlation with LES pressure reduction by POEM. Maybe it has nothing to do with the condition of muscle tissue on LES, but myotomy leads to lower LES pressure.

In conclusion, we clearly show that POEM can improve esophageal empting by lowering UES and LES pressure, thus significantly remit dysphasia. In addition, other endoscopic treatment before POEM and muscular layer myotomy did not affect the improvement of LES pressure. On the other hand, the myotomy of longitudinal muscles did not ascend LES relaxation. Besides, age and duration of symptoms has no linear correlation with LES pressure reduction by POEM. These results are promising and suggest favorable long term clinical results. Larger, clinical studies with long-term follow-up will determine whether absence of peristalsis will become a concern for POEM treatment. Longer follow-up of randomized studies comparing POEM with pneumatic dilation or LHM should be done before accepting POEM as a best treatment option for achalasia.

## Supporting Information

S1 Checklist TREND checklist. (DOC)

S1 Protocol Study protocol. (DOC)
