# Supplementary material for: Peroral Endoscopic Myotomy Can Improve Esophageal Motility in Patients with Achalasia from a Large Sample Self-Control Research (66 Patients)
Source: PLoS One. 2015 May 18;10(5):e0125942. doi: 10.1371/journal.pone.0125942 (PMC4436219; doi:10.1371/journal.pone.0125942)
Supplement: S1 Checklist — (DOC) [file pone.0125942.s001.doc]

**TREND Checklist**

| Paper Section/Topic | Content |
| --- | --- |
| Title and abstract | Peroral endoscopic myotomy can improve esophageal motility in patients with achalasia from a large sample self-control research (66 patients) |
| Background | Peroral endoscopic myotomy(POEM) as a new approach to achalasia attracts broad attention. |
| Methods |  |
| Paticipants | We screened out 205 patients with achalasia diagnosed definitely and then underwent POEM from December 2012 to April 2014 at our Digestive Endoscopic Center by Linghu EQ. Exclusion criteria included previous Heller myotomy in surgery, malignant or pre-malignant esophageal lesions. |
| Inverventions | All patients had been underwent the procedure of POEM which can be generally divided into four steps, including entry incision on mucosa, establishment of the tunnel on submucosa, myotomy and sealing of the entry incision. And 66 patients had complete HRM pre and pro POEM in our hospital.Mean follow-up period was 5.6 months. |
| Objectives | To evaluate the results with esophageal motility after POEM through the first large sample clinical research. |
| Outcomes | Upper esophageal sphincter pressure(UESP) and lower esophageal sphincter pressure (LESP) were calculated as the mid-expiratory pressure at the respiratory inversion point. Upper esophageal sphincter residual pressure(UESRP) and lower esophageal sphincter residual pressure(LESRP) were defined as the minimum pressure recorded in the LES during swallowing.  Data were compared before and within 1 year after POEM. |
| Sample size | We have a self-control research with all patients (205 in total) who underwent POEM from 2010 to 2014 at our Digestive Endoscopic Center, 66 patients of which underwent high resolution manometry(HRM) before and after POEM in our motility laboratory. |
| Assignments | Shuangzhe Yao completed data statistics,data analysis and paper writing.Enqiang Linghu operated POEM procedure of all patients and contributed to the conception of the study. Xiangdong Wang，Jiangyun Meng，technicians in Digestive Endoscopic Center， contributed significantly to assistance with POEM procedure.  Lihua Peng,Xiaoxiao Wang,doctors in motility laboratory,contributed significantly to performance of HRM for all patients. |
| Results |  |
| Paticipants flow | As a self-control study, all patients signed the consent form.  We screened out 205 patients with achalasia diagnosed definitely and then underwent POEM from December 2012 to April 2014 at our Digestive Endoscopic Center by Linghu EQ. Exclusion criteria included previous Heller myotomy in surgery, malignant or pre-malignant esophageal lesions.  Among them, 59 patients had high resolution manometry(HRM) in other Digestive Endoscopic Center, while 72 patients did not agree with HRM before POEM because of price and necessity. As a result, 74 patients have integrated HRM before and 66 patients have also after POEM. There were 34 male and 32 female patients with a mean age of 44.6 years (range 14 to 76). Mean follow-up period was 5.6 months. |
| Baseline data | The data about HRM before POEM |
| Numbers | HRM was performed in 66 patients pre and pro POEM in our motility laboratory. Mean pre-POEM UESP was 61.618 mmHg, with a standard deviation (SD) of 23.628, while mean post-POEM UESP was 57.403 mmHg with an SD of 22.911 (P > 0.05). In addition, mean pre-POEM UESRP was 16.468 mmHg, with a SD of 21.672, while mean post-POEM UESRP was 8.982 mmHg with an SD of 7.601 (P < 0.001).  Mean pre-POEM LESP was 34.678 mmHg, with a SD of 14.908, while mean post-POEM LESP was 16.612 mmHg with an SD of 8.671 (P < 0.001). In addition, mean pre-POEM LESRP was 27.459 mmHg, with a SD of 10.719, while mean post-POEM LESRP was 11.313 mmHg with an SD of 6.792 (P < 0.001).  11 of 66 patients (16.7%) had undergone other prior endoscopic treatment (endoscopic dilation or botulinum toxin injection), while 55 of 66 patients (83.3%) undergone POEM as primary treatment. There are no significant differences (P > 0.05) on the reduction of LESP and LESRP respectively between these two groups.  32 of 66 patients (48.5%) had the myotomy for circular muscles only and 34 patients (51.5%) had the myotomy of circular muscles and longitudinal muscles. Statistically, the differences between the two groups are not significant (P > 0.05) on the reduction of LESP and LESRP respectively. |
| Outcomes | 1.POEM clearly relieved the symptoms related to dysphagia by lowering the pressure of upper esophageal sphincter(UES) and lower esophageal sphincter (LES),however,underlying esophageal dysmotility on esophageal body persisted.2.Other endoscopic treatment before POEM did not affect the impovement of LES pressure.3.The myotomy of longitudinal muscle did not ascend LES relaxation. |
| Adverse events | The POEM procedure was safely performed in all patients. No technical difficulties occurred perioperatively. All patients were discharged after 7 days of hospitalization. |
| Discussion | The inviolable anatomic integrity on LES was destroyed through POEM, so the incomplete relaxation of the LES was improved when swallowing after POEM. Since the impaired inviolable anatomic integrity on LES, however, post-POEM gastroesophageal reflux remains a major problem (up to 46% in one study), the glasses style anti-reflux myotomy, which retains about 1 cm of longitudinal muscle, is expected to achieve a best result to prevent the reflux after POEM.  We can give a prediction that the symptom alleviation rate in short and medium term is excellent. However, with the formation of granulation in LES incision and the persistence of original neurogenic etiology, the recurrence rate in long term needs more follow-up studies.  When food was accumulated in esophagus of patients with achalasia, as a result, the UES was contracted compensatorily in order to avoiding the food reflux while swallowing. After POEM, with the relaxation of LES, the compensatory contraction was released. Consequently, the UESRP was reduced significantly. As for the UESP, we believe the reason for no decreasing obviously (P > 0.05) was short duration of follow-up post-POEM, the functional recovery of UES has not yet achieved completely.  The impairment of peristalsis was still persisted after POEM within one year. Maybe the improvement on peristalsis is difficult to achieve by the therapies acting on LES. On the other hand, we consider that the sensitivity of visceral perception could be decreased with LES pressure relaxed, even if the impairment of peristalsis was still persisted, the dysphagia was improved after POEM. Larger studies with long-term follow-up are needed to explain these findings.  the impairment of peristalsis was still persisted after POEM within one year. Maybe the improvement on peristalsis is difficult to achieve by the therapies acting on LES. On the other hand, we consider that the sensitivity of visceral perception could be decreased with LES pressure relaxed, even if the impairment of peristalsis was still persisted, the dysphagia was improved after POEM. Larger studies with long-term follow-up are needed to explain these findings.  longitudinal muscles of the esophagus play an important role in the physiology of motility disorders, and recent studies suggest that longitudinal muscle contraction of the esophagus induces LES relaxation and even possibly improves peristalsis of the esophagus. |
