# Supplementary material for: Peroral Endoscopic Myotomy Can Improve Esophageal Motility in Patients with Achalasia from a Large Sample Self-Control Research (66 Patients)
Source: PLoS One. 2015 May 18;10(5):e0125942. doi: 10.1371/journal.pone.0125942 (PMC4436219; doi:10.1371/journal.pone.0125942)
Supplement: S1 Protocol — (DOC) [file pone.0125942.s002.doc]

**Protocol**

**Title: A prospective randomized trial to compare the effect of POEM, BTI and BD for achalasia**

**Study institution：**Digestive Endoscopic Centre, Department of Gastroenterology and Hepatology. The Chinese PLA General Hospital.

**Source(s) of funding**：Self-funded.

**Study leader：** Enqiang Linghu

**Study leader's E-mail：**[linghuenqiang@vip.sina.com](mailto:linghuenqiang@vip.sina.com)

S**tudy leader's telephone：**+86 10 68182255

**Study leader's fax：**+86 10 68154653

**Study leader's address**：Digestive Endoscopic Centre, Department of Gastroenterology and Hepatology, No.28, Fuxing Road, Beijing, China.

**Background:**

Botulinum toxin injection (BTI), Balloon dilation (BD) and Heller myotomy (HM) are the three commonly used treatments for achalasia.BTI was confirmed to have a lower symptom remission rate than BD and HM. However,some patients would prefer to choose BTI as their first-line treatment because of its less invasiveness,better endurance,lower complication rate and shorter recovery time compared with BD and HM.And in addition,BD or HM could be a rescue therapy once TBI failed. Randomized studies comparing efficacy between BD and HM had different results and whether HM was superior to BD still remains controversial. In 2010,POEM was first introduced to clinical use by Inoue et al. and by now has been reported to have satisfactory short term efficacy by several other studies from different countries. However, complications of POEM like mucosal perforation, pneumothorax and pneumoperitoneum were not uncommon. Even seemed as a promising treatment, role of POEM in treatment for achalasia still needs to be eventually determined by randomized studies and yet no randomized studies have been reported. So, we compared efficacy and safety among POEM,BTI and BD in a randomized manner in this study.

**Aim:** To compare the effect of POEM, BTI and BD for achalasia

**Approved No. of ethic committee：**2011013-004

**Name of the ethic committee：**Committee of medical ehthics of the Chinese PLA General Hospital.

**Study ailment：**Achalasia

**Study type：**Interventional

**Study phase：**Polit

**S**t**udy design：**Randomized parallel control

## Inclusion criteria: (1) Clinical diagnosis of achalasia by an physician according to dysphagia，food reflux and retrosternal discomfort. (2) High resolution manometry shows achalasia according to losing peristalsis and last  [high](javascript:void(0);) [pressure](javascript:void(0);) in LES. (3) Esophagus barium meal examination shows achalasia according to rhamphoid  cardia and esophageal distension.

## (4)Endoscopy shows achalaisia according to food remains in esophagus and cardiac closed but endoscopy can pass, meanwhile exclude cancer.

**Exclusion criteria：(**1) Severe achalasia with sigmoid esophagus on barium esophagogram; (2) Having been treated by POEM,BTI（over twice）or BD（over twice）.

**Recruiting time：**From 2011/11/01 to 2012/12/01.

**Sample size and interventions：**15 patients in each of the 3 groups.

**Countries of recruitment and research settings:** Chinese PLA General Hospital,Beijing,China.

**Interventions**

The procedure of POEM included several steps. First, a 1.2 cm long transverse entry incision was made at about 8 cm proximal to the gastroesophageal junction (GEJ) in the right side of the esophagus with a Dual knife (KD-650Q, Olympus, Japan) after submucosal injection of about 4 to 6 ml saline solution with some drops of methylene blue. Second, a submucosal tunnel was established with its distal end reaching 3 cm below the GEJ using a Dual knife, a Hook knife (KD-620LR, Olympus, Japan) or a triangle-tip knife (KD-640L, Olympus, Japan). Third, the inner circular myotomy was completed from 5 cm above GEJ to 2 cm below GEJ by a Dual knife or a triangle-tip knife. Finally, the entry incision was closed by hemostatic clips. During the procedure of POEM, the patient was under general anesthesia in a left lateral position and carbon dioxide gas was used for insufflation.

During the procedure of BTI, a total of 100 units of botulinum toxin (Lanzhou institute of biological products, China) was injected into muscularis propria at the level of LES at one time by injecting 25 units of toxin into each of the four quadrants of the LES.

BD was performed as a single-procedure in the patients. The Rigiflex pneumatic dilation balloon (Boston Scientific, USA) used was 30 mm in diameter with its maximum pressure up to 12 PSI, which was maintained for 60 seconds after gradually reaching maximum pressure during dilation under direct endoscopic vision.

**Peri-intervention management**

Patients were fasted for 48h before treatment. For patients undergoing POEM, endoscopy was performed before anesthesia to ensure that no food residues exist in the esophagus and the stomach.

After treatment, patients were given close observations for any possible complications in the ward. Chest and abdomen X ray were performed within 2 hours after POEM and BD for early detection of possible pneumothorax, pneumomediastinum or pneumoperitoneum. After POEM and BD, patients were fasted for 3 days, and then given ﬂuid foodand gradually back to a normal diet. Patients undergoing BTI were fasted for 6 hours after treatment.

**Index:** Primary outcome is symptom remission rate and secondary outcomes include complication, lower esophageal sphincter pressure (LESP) and maximum esophageal width by barium swallow.

**Participant age:**16 to 70 years old.

**Gender:** Both.

**Method of generation of the allocation sequence：**Computer-generated

**Sign the informed consent：**Yes.

**Length of follow-up:**12 months.

**Statistical method：**Analysis of variance and Chi-square test.

**Data collection Institution：**The Chinese PLA General Hospital

**Data management Institution：**The Chinese PLA General Hospital

**Data analysis Institution：**The Chinese PLA General Hospital
